# Anesthetic management of a Rett syndrome patient with apnea and epilepsy: a case report

**DOI:** 10.1186/s40981-018-0169-y

**Published:** 2018-04-18

**Authors:** Yuka Motomura, Masafumi Idei, Hitoshi Sato, Takahisa Goto

**Affiliations:** 1 Department of Anesthesiology, Yokohama City University Hospital, 236-0004 3-9 Fukuura, Kanazawa-ku, Yokohama-shi, Kanagawa-ken Japan; 2 Department of Anesthesiology, Yokohama City University Medical Center, 232-0024 4-57 Urafune-cho, Minami-ku, Yokohama-shi, Kanagawa-ken Japan; 3 235-0033 602 2-2-2 Sugita, Isogo-ku, Yokohama-shi, Kanagawa-ken Japan

**Keywords:** Rett syndrome, General anesthesia, Perioperative management, Apnea, Epilepsy, Difficult airway

## Abstract

Rett syndrome, which is a progressive, central nervous system disease that is caused by a gene mutation, is known to present with various symptoms. This case is that of a 15-year-old girl who was diagnosed with Rett syndrome at the age of 2 years. Laryngotracheal isolation under general anesthesia was planned due to recurrent aspiration pneumonia. Since the patient’s nutritional status and control of convulsions were good, this was deemed an appropriate time for the surgery. Following careful preoperative evaluation of her airway, we performed oral endotracheal intubation using a video laryngoscope after rapid induction. Since postoperative pain control was important to prevent apneic attacks and convulsions, we used a multimodal analgesic regimen including carefully titrated fentanyl, acetaminophen, nonsteroidal anti-inflammatory drug, and wound infiltration with a local anesthetic. Postoperatively, the patient returned to the intensive care unit under spontaneous ventilation and followed a good course. Patients with Rett syndrome present several symptoms. Thus, several points must be considered during the preoperative evaluation, anesthetic management, and postoperative care of these patients.

## Background

Rett syndrome is a hereditary disease characterized by progressive neurological symptoms including hypotonia, involuntary movement, an autistic tendency, severe mental retardation, and epilepsy from infancy. The prevalence in Japan is reported to be 0.9 out of every 10,000 people, which is similar to that in other countries [1–3]. Most patients are girls and have a mutation in *MECP2* which is present on the long arm of the X chromosome.

In addition to neurological abnormalities, patients with Rett syndrome suffer from various symptoms including difficult airway due to movement restriction of the maxillofacial and temporomandibular joints; respiratory dysfunctions, such as apnea and pneumonia; and arrhythmias or electrocardiogram abnormalities, such as QT extensions and abnormal T waves [4]. Therefore, patients with Rett syndrome require attention to these issues during preoperative evaluation, intraoperative anesthetic management, and postoperative care. Although there are previous reports on anesthesia management of patients with Rett syndrome, few discuss all the complications that affect perioperative management. Thus, we report here the perioperative management of a patient with Rett syndrome and apnea and epilepsy. Especially, we paid attention to the timing of operation and postoperative analgesia.

## Case presentation

The patient was a 15-year-old girl (123.5 cm, 20.5 kg). She was born after a normal delivery. At the age of 1 year and 3 months, after it was noted that she was unable to walk, the patient was determined as having mental retardation. She was diagnosed with Rett syndrome at the age of 2 years and 9 months and developed epilepsy. At the age of 7, she underwent gastrostomy for gastroesophageal reflux under general anesthesia in another hospital. We planned laryngotracheal isolation under general anesthesia due to recurring aspiration pneumonia.

In our preoperative examination, trismus due to hypertonia was detected. It was difficult to force her mouth open manually, but it seemed to open upon intraoral cleaning and yawning, indicating that muscular hypertonia rather than contracture of the temporomandibular joints was the likely cause of trismus. Her respiratory rate at rest was 16 cycles per min. SpO_2_ in room air was 97%. Since her apneic attacks appeared 2–3 times a night and lasted around 20 s, non-invasive positive pressure ventilation (NPPV) was used at night alone. She had no heart disease, and electrocardiogram abnormalities such as prolonged QT or abnormal T waves were absent. Her epilepsy was well controlled with carbamazepine, zonisamide, and levetiracetam. Mutual communication was difficult due to the patients’ mental retardation, and she showed involuntary movement of her arms, joint rigidity, and scoliosis. Since her preoperative blood test showed that the levels of serum total protein and albumin were 6.1 and 3.6 g/dL, respectively, we judged that her nutritional status was relatively good considering her past course.

On the day of surgery, no premedication was administered. In addition to non-invasive sphygmomanometry, an electrocardiogram, and a SpO_2_ monitor, we used a bispectral index (BIS) monitor. The BIS level before the induction of anesthesia was 98. Fentanyl was administered intravenously in small increments of 10 μg because these patients may be sensitive to narcotics. Spontaneous ventilation was maintained after the first 10 μg; sursumvergence and weakened spontaneous ventilation were observed after 40 μg was administered. Thereafter, anesthesia was induced with 20 mg of propofol intravenously. After confirming the loss of the eyelash reflex, we initiated 1% sevoflurane administration and ensured that ventilation with a mask was possible. Then, 15 mg of rocuronium was administered intravenously. We initiated the intubation maneuver after confirming that the train-of-four (TOF) level was 0 (from 4). The degree of mouth opening observed was 2 breadths of a finger. Her larynx was visualized using a video laryngoscope (McGRATH™, Covidien, Dublin, Ireland) with BURP (Cormack grade II). The trachea was intubated with a spiral tube (6.0 mm inside diameter) without resistance.

Anesthesia was maintained with air, oxygen, sevoflurane, remifentanil, and fentanyl. Sevoflurane and remifentanil were administered at 1.5% of end-tidal concentration and at 0.15–0.2 μg/kg/min during the operation, respectively. The BIS level was approximately 40. In addition to the adjustment of remifentanil, fentanyl was administered several times (at a dose of 10 μg each time) to prevent the excessive rise in blood pressure. Rocuronium was added with reference to TOF, till the operation was completed. A total of 100 μg fentanyl was administered intravenously. For postoperative analgesia, 20 mg of flurbiprofen and 300 mg of acetaminophen were administered intravenously. The surgical wound was infiltrated with 9 mL of 0.25% ropivacaine. Since the TOF level at the end of surgery was 85%, we administered 40 mg of sugammadex intravenously to reverse the neuromuscular relaxant. After discontinuing remifentanil, spontaneous ventilation resumed immediately. Once we confirmed that there were no abnormal findings in the chest X-ray, administration of sevoflurane was stopped. When the BIS recovered to 60, body movement appeared and the cough reflex at the endotracheal suctioning was confirmed. Hypertonia was occasionally detected by stimulation, but disappeared spontaneously. Clear apneic attacks and systemic convulsions were absent. Since the tidal volume was 200–250 mL and respiratory rate was 15–20 times per min stably, we considered her respiratory status stable and mechanical ventilation was weaned. A heat-moisture exchanger (HME) was attached for the tracheotomy while 1 L of oxygen was administered per min.

Since her SpO_2_ was constantly 99–100%, she was transferred to the ICU under spontaneous ventilation. She followed a good postoperative course without any complications (such as exacerbated apneic attacks or systemic convulsions) and was discharged 4 weeks later.

## Discussion

Patients with Rett syndrome require sufficient preoperative evaluation. In particular, the evaluation of respiration, including the evaluation of the airway, arrhythmia, electrocardiogram, epilepsy, and nutritional status are important. Regarding airway and respiration, attention should be paid to the excursion restrictions of the temporomandibular joint with myotonia, trismus, apneic attack, pneumonia, and presence of ventilatory dysfunction due to scoliosis. Patients with Rett syndrome may also have electrocardiogram abnormalities, such as prolonged QT and abnormal T wave, bradyarrhythmia, and sinus dysfunction, all of which may lead to sudden death [5]. Great attention is required when using a drug that induces QT prolongation during surgery. Epilepsy is seen in 80% of patients with Rett syndrome, of whom 46% are drug-resistant [6, 7]. These patients should also be regarded at risk of perioperative epilepsy. The frequency of epileptic attacks and whether epilepsy is well controlled must be evaluated sufficiently. Evaluation of nutritional status is also important because weight loss and exacerbation of nutritional status are involved in prognosis [8].

Although apneic attack was detected in the present case, the patient did not have inflammation and fever to indicate active aspiration pneumonia preoperatively. She did not present with electrocardiogram abnormalities. Her epilepsy was well controlled with oral anticonvulsant medication and her nutritional status was relatively good. Therefore, we determined that the timing of her operation was appropriate.

Patients with Rett syndrome reportedly have individual variability in sensitivity to anesthetic agents and muscle relaxants [9, 10]. Therefore, it is strongly recommended that the BIS monitor and muscle relaxation monitor be used to avoid both insufficient anesthesia and delayed awaking. Towards the end of an anesthetic and during the postoperative period, insufficient analgesia and premature awakening may induce apneic attacks and convulsions in these patients. Conversely, generous doses of narcotics may cause respiratory depression because these patients may be sensitive to narcotics. Therefore, a multimodal technique would be valuable to provide postoperative analgesia. Since analgesic evaluation in patients with Rett syndrome may be difficult due to difficulty in communication, close patient observation and careful titration of analgesic drugs are required. Table 1 outlines the perioperative problems and evaluation items corresponding to the current patient with Rett syndrome.

**Table 1:** Perioperative problems, evaluation items, and correspondences of Rett syndrome patient

	Perioperative problem of Rett syndrome patients	Preoperative evaluation	Intraoperative management	Postoperative management
Central nervous system	Intellectual disability Spasm Autonomic disturbances Variability in sensitivity to anesthetic agents	Mutual understanding may be difficult Control of epilepsy Adjustment of Dosage of antiepileptics	Consider premedication Appropriate analgesia/anesthesia Avoid drugs that induce spasm Prevent hypotension during induction Prevent delayed emergence from anesthesia Appropriate monitoring (BIS/TOF)	Avoid seizure with adequate multimodal analgesia Early resumption/adjustment of anticonvulsants Appropriate monitoring
Airway	Micrognathia, Trismus Technical difficulties of intubation	Evaluate technical difficulties of intubation	Prepare video laryngoscope or bronchofiber Consider awake intubation	
Respiratory system	Apnea/abnormality of respiratory pattern Pneumonia Restrictive ventilatory impairment	Preoperative examination Evaluate pneumonia with blood test, X-ray	Avoid apnea with adequate analgesia	Close monitoring of respiratory conditions Physical therapy to avoid respiratory complications NPPV
Circulatory system	QT prolongation Arrhythmia Bradycardia Structural cardiac defects are rare	Electrocardiogram Echocardiography	Avoid drugs that prolong QT Consider cardiac pacing Monitoring electrocardiogram	Management under ECG monitor
Nutrition	Malnutrition Hypoalubuminemia Electrolyte abnormalities	Evaluate nutritional status, surgical indication Correct nutritional status, electrolyte abnormalities		Early enteral nutrition
Musculoskeletal system	Scoliosis		Appropriate positioning and decompression to prevent bedsores	

## Conclusions

We conducted general anesthesia management of a patient with Rett syndrome with central nervous symptoms using appropriate preoperative evaluation, optimal anesthesia management, and monitoring, without complications. A detailed anesthetic plan is required in the perioperative management of patients with Rett syndrome because several points must be considered during preoperative evaluation, intraoperative anesthetic management, and postoperative care.

## Abbreviations

BIS: Bispectral index
BURP: Backward upward rightward pressure
HME: Heat-moisture exchanger
ICU: Intensive care unit
NPPV: Non-invasive positive pressure ventilation
TOF: Train-of-four
